# Purification, antioxidant activities, encapsulation, and release profile of total flavonoids in Peony seed meal

**DOI:** 10.1002/fsn3.2731

**Published:** 2022-01-21

**Authors:** Lixia Zan, Wangting Song, Weiwei Wang, Gang He, Xinsheng Li, Jinjin Pei

**Affiliations:** ^1^ College of Bioscience and Bioengineering Shaanxi University of Technology Hanzhong China; ^2^ College of Life Sciences Northwest University Xi’an China

**Keywords:** antioxidant activity, complex coacervation, microcapsule, peony seed meal, total flavonoids

## Abstract

As potential biomass resources, biomass waste products have been considered worldwide in recent decades. Peony seed meal (PSM) is a kind of agricultural resource waste containing polyphenols, in particular flavonoids. In this study, the total flavonoids of PSM were extracted and purified by AB‐8 macroporous resin (MR), the antioxidant activities of three extract fractions were evaluated, and the total flavonoids were encapsulated with alginate and chitosan by the complex coacervation method. After purification, the yield of total flavonoids was 11.32% and the content in the product increased to 42.89% ± 2.66. The antioxidant activities of three fractions on ^·^OH, DPPH, and ABTS assays exhibited the following descending order: ethanol elution fraction (ELF) > ethyl acetate extract fraction (EAF) > ethanol extract fraction (EEF). The single‐factor assay showed that the encapsulated total flavonoid microcapsules (EFMs) were prepared with a chitosan concentration of 10 mg/ml, a sodium alginate concentration of 30 mg/ml, a calcium chloride concentration of 50 mg/ml, a ratio of sodium alginate to total flavonoids of 1:3, a flavonoid concentration of 40 mg/ml, and an encapsulation yield of 80.7%. Most microcapsules are smooth‐faced, spherical and uniform in size ranging from 2 to 3 mm in diameter. In vitro release studies suggested that the EFM was stable at pH 1.2 and dissolved at pH 7.5. The result indicated that the EFM is worthy for the development of functional foods and supplements, and PSM could be a potential resource in the food and pharmaceutical industries.

## Graphical abstract

This present study focused on the purification, antioxidant activities, encapsulation, and release profile of total flavonoids in Peony seed meal. The result indicated that the encapsulated total flavonoid microcapsules are worthy for the development of functional foods and supplements, and Peony seed meal could be a potential resource in the food and pharmaceutical industries.

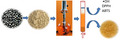

## 1 INTRODUCTION

*Paeonia ostii*, that is one of the wild species of tree peony, was authorized as a new food resource by the National Health and Family Planning Commission of the People's Republic of China in November 2013 (Zhang et al., 2017). *Paeonia ostii* seed oil is well known as a functional edible oil because of riching in α‐linolenic acid (>40%) and polyunsaturated fatty acids (>90%). However, with the increasing market demand of *Paeonia ostii* seed oil, agroindustrial wastes, and food by‐products, a large amount of PSM is produced in industrial production (Wang, Li, et al., 2019a). Previous research on Peony has focused on the seed oil and the flavonoid content (Li et al., 2009; Zhang et al., 2017). PSM has been previously investigated for its proteins and peptides (Zhang et al., 2017, 2019, Wang et al., 2019b), but few studies were conducted on flavonoid compounds present.

Flavonoids are equipped with potential health benefits such as antioxidant, antiviral, antiallergenic, and anti‐inflammatory properties (Li et al., 2009, Zhang et al., 2017). Nonetheless, their beneficial properties can be affected by a variety of factors including light, heat, oxygen levels, and pH on account of their instability (Peanparkdee & Iwamoto, 2020). After oral administration, the flavonoids degrade in gastric acid, resulting in a reduction of the health benefits. Therefore, increasing the stability of flavonoids in this environment could greatly improve their potential application.

Microencapsulation is a technology that involves packaging sensitive elements in a protective coating film to prevent the constituents from an adverse environment. The complex conservation method is a kind of microencapsulation technique that exhibits a high loading capacity, low temperature, and less thermal degradation reaction, as a result of which it can better control the release of active substances (Gouin, 2004; Ozkan et al., 2019).

We extracted and purified total flavonoids of PSM, evaluated the antioxidant activities of the extracts, and encapsulated the total flavonoids to improve the stability. Additionally, we analyzed the in vitro release behavior of the microcapsules. Ultimately, this study will provide sufficient experimental evidence for the further development and utilization of total flavonoids of PSM.

## 2 MATERIAL AND METHODS

### 2.1 Materials and chemicals

PSM was purchased from Tianhua Ecological Agriculture Development Co., Ltd. in October 2019. Hydrogen peroxide 30% was obtained from Tianli Chemical Reagent Co., Ltd. Salicylic acid was purchased from Baishi Chemical Co., Ltd. Ferric sulfate, ascorbic acid (Vc), and acetic acid were purchased from Xilong Chemical Co., Ltd. Hydrochloric acid and sulfuric acid were obtained from the Beijing Chemical Factory. Ethanol, ethyl acetate, sodium nitrite, and sodium hydroxide were purchased from Beilian Fine Chemicals Co., Ltd. DPPH and ABTS were obtained from Fuyang Manlin Biotechnology Co., Ltd. Chitosan and sodium alginate were purchased from Sinopharm Chemical Reagent Co., Ltd. Calcium carbonate was purchased from Fuchen Chemical Reagent Factory. Calcium chloride was obtained from Yongsheng Fine Chemical Co., Ltd. Potassium persulfate was purchased from Shengao Chemical Reagent Co., Ltd. Rutin was purchased from Nanjing Aoduofuni Biotechnology Co., Ltd. Aluminum nitrate was purchased from Chengdu Cologne Chemical Co., Ltd. AB‐8 MR was purchased from Guangfu Fine Chemical Research Institute. Water was purified by the Milli‐Q system (UPH‐II‐20T; Ulupure).

### 2.2 Preparation of total flavonoids

#### 2.2.1 Extraction of total flavonoids

Total flavonoids of PSM were extracted according to the previous procedure (Li et al., 2002) with some modifications as follows: The defatted PSM powder was added with a material–liquor ratio of 1:20 (m/v) with 75% ethanol and extracted thrice in ultrasound bath (SB‐5200DT; Xinzhi) at 100 W for 1 h. The combined ethanol extract (CEE) was filtered and concentrated by vacuum evaporation which reduced the volume to 30 ml at 40℃ (R206; Senco). Then, the concentrate was extracted three times with the same volume of ethyl acetate, and EAF was obtained by drying the ethyl acetate fraction. The EEF was obtained by drying the CEE, and the crude flavonoid solution (CFS) was obtained by adding 1,000 ml of Milli‐Q water to 1,000 mg of CEE.

#### 2.2.2 Purification of total flavonoids

To enrich and purify the total flavonoids, CFS was loaded into the AB‐8 MR column (3 × 60 cm) at a flow rate of 2 ml/min. After adsorption equilibrium, it was eluted with four bed volumes of water and 2 bed volumes of a 30% ethanol in turn at a flow rate of 2 ml/min, and then, the total flavonoids were eluted with 50% ethanol. The ELF was obtained by drying the 50% ethanol eluent.

### 2.3 Determination of total flavonoid content

Total flavonoid contents were determined by sodium nitrite–aluminum nitrate colorimetric method (Hao et al., 2018) as described previously, with modifications stated as below: 5 ml of 75% ethanol solution of the rutin or flavonoids was mixed with 0.5 ml of 5% NaNO_2_ solution for 6 min, and then, 0.5 ml of 10% Al(NO_3_)_3_ and 3 ml of 4% NaOH were added and mixed fully for 15 min. The absorbance of the mixture was measured at 510 nm with an ultraviolet‐visible spectrophotometer (U‐3900H; Hitachi) in accordance with its standards. A calibration curve of rutin was prepared as described by the previous method (Meng et al., 2013). Three replicates were prepared per sample.

### 2.4 Antioxidant activities of total flavonoids

#### 2.4.1 Hydroxyl radical scavenging capacity

The antioxidant activity was measured by a hydroxyl radical scavenging assay involved in the Fenton reaction, according to a previous method (Wang, Zhao, et al., 2019b). The mixture was prepared by mixing 1 ml of the sample solution or Vc solution, 1 ml of 9 mM FeSO_4_ solution, 1 ml of 9 mM salicylic acid/ethanol solution, and 1 ml of 8.8 mM H_2_O_2_ and then incubated in water bath at 37℃ for 30 min. The spectral absorption of the mixture was measured at 510 nm.

The hydroxyl radical clearance rate was calculated according to the following equation:

$$\begin{array}{c} {}^{\cdot }\text{OH scavenging capacity}\;\left(\%\right)=1‐A_{\text{sample}}/A_{\text{control}}\times 100\% \end{array}$$

where *A*_sample_ and *A*_control_ refer to the spectral absorption of the extract or standard and the blank control, respectively. All tests were run in triplicate to obtain the mean value.

#### 2.4.2 DPPH radical scavenging capacity

The DPPH‐free radical scavenging capacity was determined according to the method described previously with some modifications (Gong et al., 2016). The reaction mixture was prepared by mixing 2 ml of 4% DPPH ethanol solution and 2 ml of sample solution or Vc solution. The mixture was kept at room temperature for 30 min and tested at 517 nm to obtain the spectral absorption.

The DPPH scavenging capacity was counted according to the following formula:

$$\begin{array}{c} \text{DPPH scavenging capcity}\;\left(\%\right)=1‐A_{\text{sample}}/A_{\text{control}}\times 100\% \end{array}$$

where *A*_sample_ and *A*_control_ denote the spectral absorption of the extract or standard and the blank control, respectively. All tests were run in triplicate to obtain the mean value.

#### 2.4.3 ABTS radical scavenging capacity

The ABTS radical scavenging activity was determined in reference to a previous method (Villano et al., 2004). The stock solution was prepared by mixing equal volumes of 7.4 mM ABTS solution and 2.45 mM of potassium persulfate solution, and being left to a dark reaction for 16 h at room temperature. Then, the ABTS solution was obtained by diluting the stock solution with absolute ethyl alcohol, until the absorbance of the working solution was 0.70 ± 0.02 at 734 nm.

The reaction mixture was prepared by mixing 3.9 ml of ABTS solution and 0.1 ml of sample or Vc solution for 6 min at room temperature, and the spectral absorption was measured at 734 nm. The scavenging rate of ABTS radicals was calculated as the following equation:

$$\begin{array}{c} \text{ABTS scavenging capacity}\;\left(\%\right)=1‐A_{\text{sample}}/A_{\text{control}}\times 100\% \end{array}$$

where *A*_sample_ and *A*_control_ represent the spectral absorption of the extract or standard and the blank control, respectively. All tests were run in triplicate to obtain the mean value.

### 2.5 Preparation of EFM

The total flavonoids were encapsulated with alginate and chitosan by the complex coacervation method with slight modifications (Coulombier et al., 2020; Koppolu et al., 2014). 50ml chitosan solution was added to equal amount of calcium chloride solution, and the pH was adjusted to 3.5 with acetic acid. The solution was mixed at 100 rpm at 25℃ with a magnetic stirrer (SH‐3; Taisite) to obtain the mixture of chitosan and calcium chloride (CCS). The mixture of total flavonoids and sodium alginate solution was evenly dripped into CCS with an infusion pump and stirred slowly for 20 min. The EFM was obtained.

### 2.6 Effects of formulation factors on encapsulation yield

The effects of formulation factors on the encapsulation yield were evaluated by single‐factor experiment, including chitosan concentration (2.5–12.5 mg/ml), sodium alginate concentration (10–50 mg/ml), calcium chloride concentration (10–90 mg/ml), ratio of sodium alginate to total flavonoids (1:1–1:5), and total flavonoid concentration (10–50 mg/ml).

### 2.7 Encapsulation yield of EFM

The encapsulation yield of EFM was measured using the methods described previously with some modifications (Cai et al., 2019). 500 mg of microcapsules was washed with 5 ml of ethanol, and supernatant was collected to determine the surface flavonoid content of the microcapsules (A).

500 mg of microcapsules was extracted with 5 ml of ethanol in ultrasound bath for 10 min and then filtered to determine the total flavonoid content in supernatant (B). The encapsulation yield of the total flavonoids was counted using the following equation:

$$\begin{array}{c} \text{Encapsulation yield}\;(\%)=(1‐A/B)\times 100\% \end{array}$$

### 2.8 In vitro release of EFM

The simulated gastric and intestinal fluids were prepared according to a previous method with some modifications (Adejoro et al., 2019). The simulated gastric fluid was prepared by adding 9 ml of 1 M HCl in 1 L water, and the final pH was then adjusted to 1.2. The simulated intestinal fluid was prepared by adding 6.8 g KH_2_PO_4_ in 250 ml water and 380 ml of 0.1 M NaOH. Finally, the solution was diluted to 1 L with water and adjusted to pH 7.5.

In vitro release of EFM under gastric and intestinal conditions was monitored by incubating 100 mg of microcapsule in 40 ml simulated gastric and intestinal fluid and continuously stirring at 60 rpm at 37℃ for 3 hr. Aliquots of 1 ml were removed for analysis at 10, 20, 30, 40, 50, 70, 100, 120, 150, and 180 min after incubation. Aliquots were taken immediately for subsequent analysis, and the solution was replenished to the initial volume. The release of total flavonoids was monitored by UV‐Vis spectrophotometry as described above, and the release curve was drawn.

## 3 RESULTS AND DISCUSSION

### 3.1 Sample extraction and preparation

Total flavonoids of PSM were extracted and purified. The weight of EEF was 36.75 g, EAF was 15.37 g, and ELF was 5.66 g. Table 1 shows the extract yield and contents of total flavonoids of three extract fractions. The extract yield of three fractions varied from 73.5% to 11.32%. Among all the fractions, EEF has the highest extraction yield (73.5%) while the ELF had the lowest yield (11.32%). This was due to the polarity of extract solvents and procedures, with the further purification, the purity of total flavonoids in the extract increased. Therefore, this method is suitable for the extraction and purification of flavonoids in PSM. An AB‐8 MR is a kind of spherical, low‐polar polystyrene with a large surface area ranging from 480 to 520 m^2^•g^−1^, and a suitable pore diameter between 130 and 140 Å. Overall, it is a type of flavonoids adsorbent with superior performance (Zhang et al., 2018).

**TABLE 1:** Extraction yield and total flavonoid contents of each fraction

Index	EEF	EAF	ELF
Extract yield (%)	73.5	30.74	11.32
Total flavonoid contents (%)	15.60 ± 1.42	28.75 ± 1.94	42.89 ± 2.66

Note

Values were given as mean (±*SD*).

### 3.2 Antioxidant activity

#### 3.2.1 Hydroxyl radical scavenging activity

**FIGURE 1:**
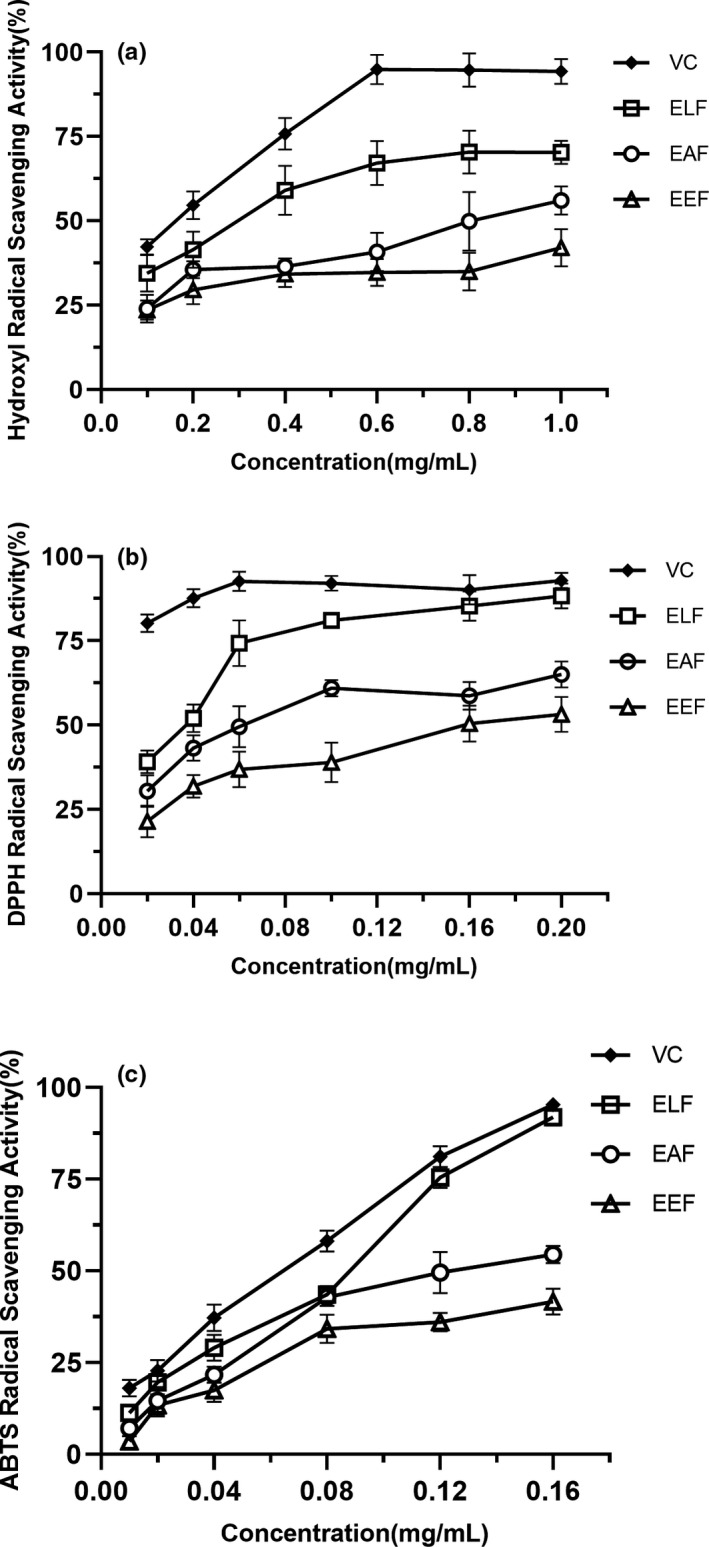
Antioxidant activities of three extract fractions; EEF, ethanol extract fraction; EAF, ethyl acetate extract fraction; and ELF, ethanol elution fraction. (a) Hydroxyl radical scavenging activity, (b) DPPH radical scavenging activity, and (c) ABTS radical scavenging activity

The hydroxyl radical is an extremely active‐free radical in biological systems, which can cause oxidative damage to biological macromolecules. Antioxidants can prevent damage to the hydroxyl radical of biological macromolecules (Sham et al., 2017). The hydroxyl radical scavenging activity of three extract fractions was shown in Figure 1a. The hydroxyl radical scavenging activity of extracts increased with an increase in concentrations. In addition, the scavenging activity of the fractions presented the tendency of reduced ability in the following order: Vc >ELF > EAF >EEF, which were 94.25%, 70.33%, 56%, and 42.05% at 1 mg/ml, respectively.

#### 3.2.2 DPPH radical scavenging activity

The DPPH^·^ is a stable free radical of a red‐purple color that is able to accept an electron or hydrogen radical to become a stable diamagnetic molecule, causing a decrease in absorbance at 515 nm (Yang et al., 2018). As such, it is widely applied to assess antioxidative properties (Gong et al., 2016). The DPPH radical scavenging activity of different fractions was exhibited in Figure 1b. At concentrations from 0.02 to 0.2 mg/ml, the DPPH radical scavenging ability of ELF ranged from 39.16% to 88.29%, that of EAF was 30.42%–65.02%, that of EEF was 21.45% to 53.20%, and that of Vc increased from 80.22% to 92.91%. With the increase in concentration of fractions, the samples' DPPH radical scavenging ability gradually increased. ELF exhibited the strongest reducing power in the three fractions, slightly lower than Vc.

#### 3.2.3 ABTS radical scavenging activity

ABTS^·+^ as a synthetic radical is often used to determine the scavenging activity of both polar and nonpolar antioxidant compounds (Yang et al., 2018). In the ABTS test, antioxidants scavenge the blue chromophore ABTS^·+^ by either electron donation or hydrogen electron transfer, thus inducing discoloration at 734 nm (Sham et al., 2017). As shown in Figure 1c, the ABTS‐free radical scavenging activity of three extract fractions was examined. Each fraction has the different inhibitory effects on ABTS‐free radicals. The inhibition was concentration dependent, while the scavenging capability increased with sample concentration. In addition, the scavenging effect of different fractions on ABTS decreased in the order of: Vc >ELF > EAF >EEF, which were 95.31%, 91.90%, 54.48%, and 41.65% at a 0.16 mg/ml concentration, respectively.

### 3.3 Effect of factors on encapsulation yield

**FIGURE 2:**
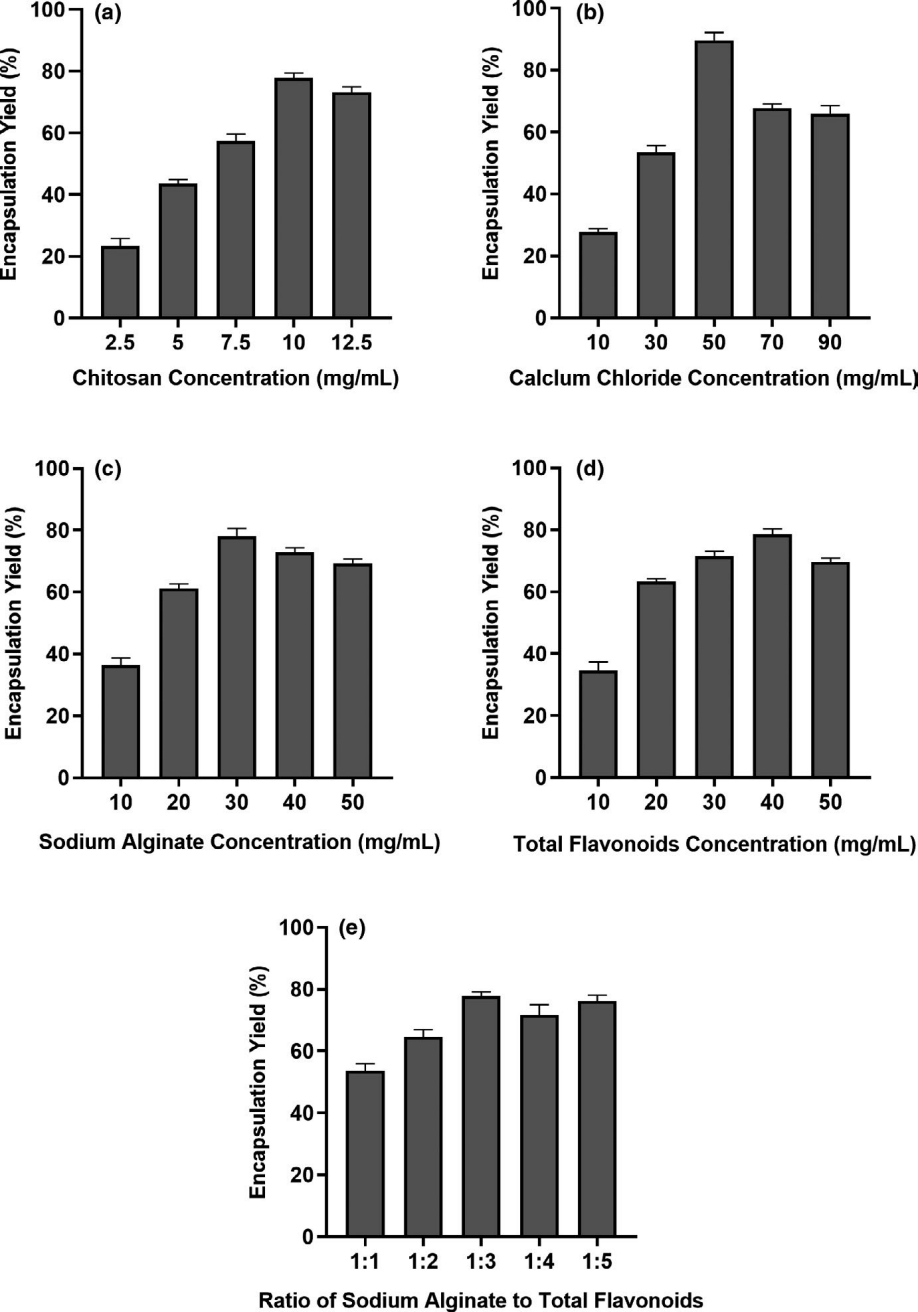
Effect of five formulation factors on encapsulation yield (a) factor with the chitosan concentration, (b) factor with calcium chloride concentration, (c) factor with sodium alginate concentration, (d) factor with total flavonoid concentration, (d) factor with the ratio of sodium alginate to total flavonoids

The encapsulation yield was an important index to evaluate the microcapsule embedding effect. As shown in Figure 2, the five formulation factors influencing the encapsulation yield were studied, such as the chitosan concentration, sodium alginate concentration, calcium chloride concentration, total flavonoid concentration, and the ratio of sodium alginate to flavonoids.

As can be seen in Figure 2a, with the increase in chitosan concentration from 2.5 to 10 mg/ml, the encapsulation yield increased and reached a maximum at 10 mg/ml, but decreased at 12.5 mg/ml. It can be seen from Figure 2b that when the calcium chloride concentration increased, the encapsulation yield increased and reached a maximum at 50 mg/ml. When the calcium chloride concentration kept increasing, the size of the microcapsules got large, the wall became thick and the encapsulation yield decreased. In Figure 2c the encapsulation yield of microcapsules first increased when the sodium alginate concentration increased from 10 to 30 mg/ml, but gradually decreased as the sodium alginate concentration increased from 30 to 50 mg/ml. As shown in Figure 2d, the encapsulation yield of microcapsules gradually increased and reached its maximum when the total flavonoid concentration increased from 10 to 40 mg/ml. As can be observed in Figure 2e, when the sodium alginate to total flavonoids increased in the range of 1:1 to 1:5, the maximum encapsulation yield was 77.78% at 1:3. Therefore, the ratio of sodium alginate to total flavonoids should be around 1:3. Moreover, under this condition, the forming effect of the microcapsule was better and the trailing phenomenon was lower.

Based on the evaluation of the effects of five formulation factors on the encapsulation yield by single‐factor experiment, the total flavonoid‐loading alginate–chitosan microcapsules were prepared with a chitosan concentration of 10 mg/ml, a calcium chloride concentration of 50 mg/ml, a sodium alginate concentration of 30 mg/ml, total flavonoids concentration of 40 mg/ml, and a ratio of sodium alginate to flavonoids of 1:3. The results of verification tests showed the encapsulation yield was 80.7%.

### 3.4 Morphology and size of EFM

**FIGURE 3:**
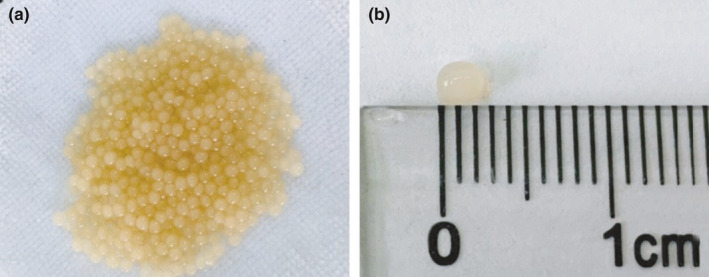
Morphology and size of encapsulated total flavonoid microcapsules (a) morphology and (b) size

Alginate–chitosan microcapsules were prepared in two steps by the complex coacervation method. First, gelation reactions led to the formation of the microcapsule frame. When the sodium alginate solution meets divalent cations, it changes from a homogeneous liquid to a gel state. Secondly, the negatively charged hydroxyl group of sodium alginate and the positively charged primary amino group of chitosan can be attracted by electrostatic interaction to form a stable polyelectrolyte membrane (Coulombier et al., 2020). As showed in Figure 3a, EFM presented well‐dispersed, pale yellow, and smooth‐faced spherical particles, with most particles being uniform in size ranging from 2 to 3 mm in diameter in Figure 3b.

### 3.5 In vitro release of EFM

**FIGURE 4:**
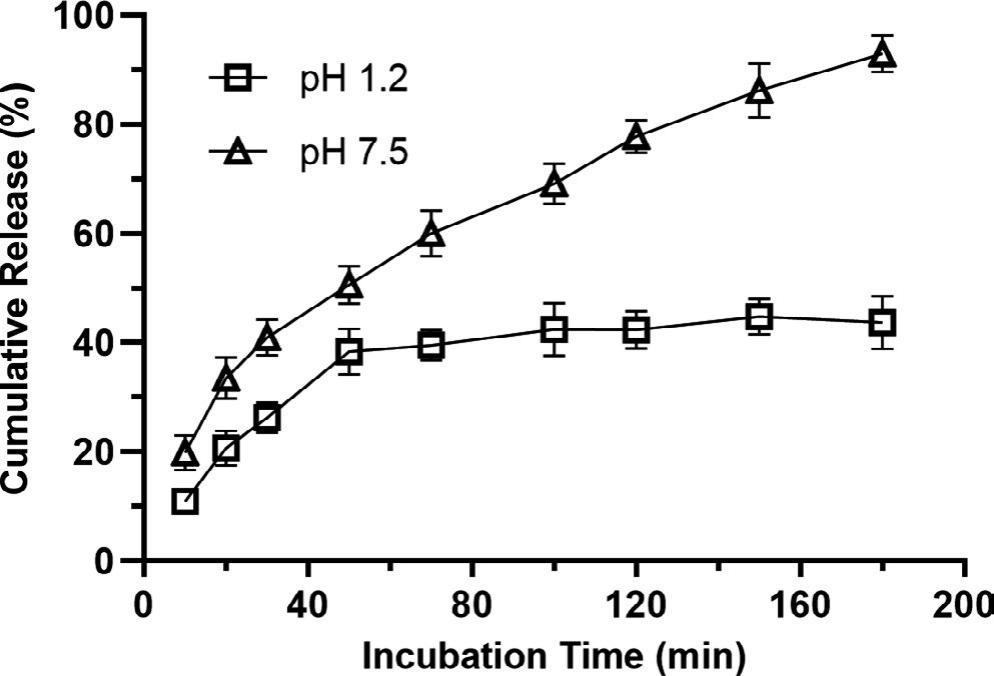
In vitro release profile of total flavonoid microcapsules

In vitro release of EFM was observed as shown in Figure 4. The percentage of total flavonoids released from microcapsules under pH 7.5 was faster than pH 1.2. A rapid release pattern appeared within the first 50 min of incubation for flavonoid microcapsules in both media. This phase was followed by a phase of gradual release from 50 to 180 min in pH 1.2, and about 43.74% of total flavonoids were released within 180 min. The release profile indicates that 50.59% and 92.97% of total flavonoids was released from the microparticles at 50 min and 180 min in pH 7.5, respectively. The results indicate that the wall material structure of EFM is more stable in pH 1.2 than in pH 7.5, which prevented the release of flavonoids from the microcapsules.

## 4 CONCLUSIONS

This present study focused on the purification, antioxidant activities, encapsulation, and release profile of total flavonoids of PSM. The results showed the purified ELF had the highest contents of total flavonoids among three fractions, which perhaps contribute greatly to the highest antioxidant activities. Additionally, alginate and chitosan were successfully used in encapsulating total flavonoids with an encapsulation yield of 80.7%. In vitro release studies suggested that the EFM was stable at pH 1.2 and dissolved at pH 7.5. It means that microencapsulation may be a suitable method for stabilizing flavonoids, which allows EFM to be retained in the stomach and released in the intestine. The result indicated that the EFM is worthy for the development of functional foods and supplements, and PSM could be a potential resource in the food and pharmaceutical industries.

## CONFLICT OF INTEREST

The authors declare no conflict of interest.

## Data Availability

The data used to support the findings of this study are available from the corresponding author upon request.
